# An Indirect Sewage Nuisance

**Published:** 1900-09-29

**Authors:** 


### AN INDIRECT SEWAGE NUISANCE.

An enormous amount of labour has been devoted by inventors to the purification of sewage, and many have been the disappointments which they have met with. For a time it was thought that if an effluent were clear it was enough, and when they saw that the streams flowing from their tanks were bright and transparent they were content. It was soon found, however, that no amount of clearness, or of freedom from odour and suspended matter, gave any guarantee that when an effluent got a few miles further down the river it would not again become offensive from renewed decomposition. It became obvious then that an effluent must be so thoroughly decomposed as to have arrived at a state of chemical equilibrium, such a state that no further decomposition shall take place, before its discharge into the river or other water into which it is to flow. Even such an effluent, however, contains a large amount of nitrogenous material which is useful as a manure, and undoubtedly the proper plan is to use it for irrigating the land, so as to reclaim from it the fertilising ingredients which otherwise would be wasted. There are places, however, where it is difficult, if not impossible, to obtain land for such a purpose, and in such cases many have thought that an effluent which is clear and thoroughly decomposed may safely be thrown away; and that although to cast it into sea or river may seem wasteful, it at least will do no harm and cause no nuisance. Nature, however, is by no means easily put in leading strings, and now the question arises whether the discharge of so rich an effluent, even though chemically it may be incapable of further decomposition, may not so encourage aquatic life, and set up so vast a growth of water weeds—which in turn must die and rot—as to give rise to a secondary nuisance almost as great as might have been caused by its immediate discharge in an unpurified state. At the British Association at Bradford a paper was contributed by Dr. Letts and Mr. Hawthorne, of Belfast, on the relation of certain sea weeds to the pollution of sea water by sewage, which tended to show that where sewage is discharged into the sea it encourages the growth of a peculiar sea weed—the ulva latissima, or sea lettuce—to such an extent that when high winds or gales prevail it is washed ashore in enormous quantities and by its decomposition causes an overpowering nuisance. In Belfast Lough the quantity thus deposited is said to be enormous, forming banks which are often several feet thick and extend for miles along the coast. The same thing occurs in a minor degree in Dublin Bay, and in regard to fresh water we have long been familiar with various forms of fungi the growth of which seems to depend on the over-rich condition of the streams and rivers in which they grow, a condition resulting from the discharge of effluents from sewage farms. The sewage problem is evidently not solved by the production of a clear or even of a stable effluent, and it may be questioned whether we shall ever be able to depart far from those natural processes which aim at imitating Nature in her ways, processes which, unfortunately, as in broad irrigation and similar arrangements, demand space, and light, and air for their due accomplishment.

